# Clinical Features of RS Virus Infection before, during, and after COVID-19 Pandemic

**DOI:** 10.3390/children11080922

**Published:** 2024-07-30

**Authors:** Natsu Ide, Kyosuke Tabata, Norihiro Tokuma, Yayoi Murano, Daisuke Yoneoka, Tomoyuki Nakazawa, Hiromichi Shoji

**Affiliations:** 1 Division of Pediatrics, Tokyo Metropolitan Toshima Hospital, Tokyo 173-0015, Japan; 2 Department of Pediatrics, Juntendo University Faculty of Medicine, Tokyo 113-0033, Japan; 3 Center for Surveillance, Immunization, and Epidemiologic Research, National Center of Infectious Disease, Tokyo 102-0071, Japan

**Keywords:** COVID-19, pandemic, RS virus infection

## Abstract

The COVID-19 pandemic has impacted the epidemiology of other infectious diseases. In particular, the respiratory syncytial (RS) virus infects almost all children during their first or second year of life. However, during the COVID-19 pandemic, many restrictions were enforced that isolated children from other children. Therefore, we hypothesized that the clinical features of RS virus infection were altered and conducted a study to evaluate these changes. This observational study included children below the age of six years who were admitted to the Tokyo Metropolitan Toshima Hospital. Their clinicodemographic data were extracted from medical records. The 369 children eligible for the study were assigned to three groups: “pre-pandemic” (group 1, n = 253); “during pandemic” (group 2, n = 77), and “post-pandemic” (group 3, n = 39). Logistic regression analysis revealed that compared to group 1, the odds ratio (OR) for oxygen use was significantly higher in groups 2 (OR 1.85. 95% confidence interval [CI] 1.06–3.23; *p* < 0.05) and 3 (OR 3.36, 95% CI 1.59–7.12; *p* < 0.01), and the use of mechanical ventilation was significantly higher in group 3 (OR 4.89, 95% CI 1.71–13.94; *p* < 0.01). This study highlights changes in the clinical features of RS virus infection during and after the COVID-19 pandemic.

## 1. Introduction

The COVID-19 pandemic has altered the epidemiology of infectious diseases [1,2]. The respiratory syncytial (RS) virus is a common pathogen that infects nearly all children at least once before they are two years of age. The severity of symptoms is highly dependent on the age of the child [3]. RS virus is transmitted through direct contact with infected droplets [3]. During the COVID-19 pandemic, many nurseries were closed, and persons avoided taking their children to crowded places. Consequently, the decrease in direct contact resulted in fewer RS virus infections. In fact, the number of children with RS virus infections declined to almost 0 at the 13th week of 2020, two months after the first COVID-19 case was detected in Japan, until the third month of 2021. This was followed by a marked increase thereafter [4]. Given that RS virus has a considerable impact on younger children [5], understanding the trends in its epidemiology and clinical features is crucial.

Non-pharmaceutical interventions, including traveling restrictions, hand hygiene, and the wearing of masks, are well-known methods to reduce the number of RS virus infections in children [6]. Although several studies have reported changes in the epidemiology of RS virus [7,8], they have focused primarily on COVID-19. However, we consider non-pharmaceutical interventions to be more important factors associated with the epidemiology of the RS virus and aim to assess the clinical features of RS virus infections based on the time periods associated with them, relative to the period of the COVID-19 pandemic.

## 2. Materials and Methods

### 2.1. Study Participants

This is an retrospective observational study using medical records. The study participants are children under six years old, who were admitted to the Tokyo Metropolitan Toshima Hospital between August, 2017 and July, 2023. The following de-identified data were extracted: the day of admission, age at admission, premature birth (yes/no), trisomy 21 (yes/no), congenital heart disease (yes/no), chest X-ray finding (pneumonia yes/no), medicine use during hospital stay (steroid, antibiotics), use of oxygen and/or mechanical ventilation (including nasal high-flow therapy), and laboratory data, such as white blood cell count (/μL), neutrophils (%), and C-reactive protein (mg/dL) level. 

**Figure 1:**
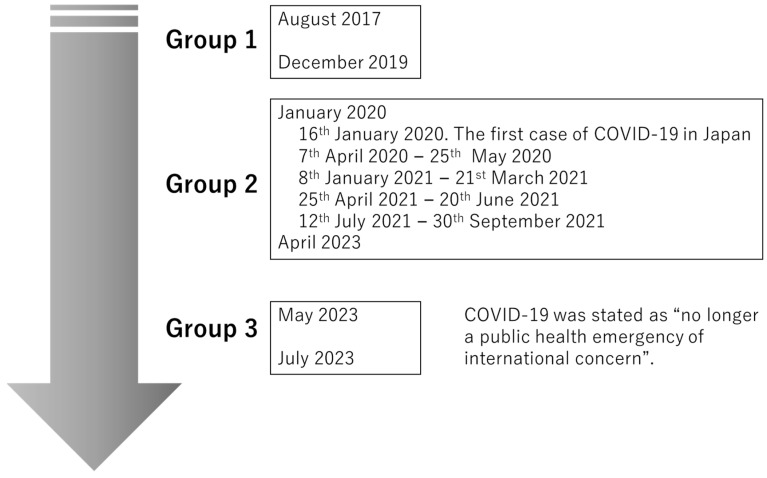
Categorization of groups in the study.

In Japan, the first COVID-19 case was reported on 16 January 2020. A state of emergency was declared from 7 April to 25 May 2020; 8 January to 21 March 2021; 25 April to 20 June 2021; and July to 30 September 2021. In May 2023, COVID-19 was declared as “no longer a public health emergency of international concern”. Participants were divided into three groups based on the time of their hospital admission: group 1 (pre-pandemic, between August 2017 and December 2019); group 2 (during pandemic, between January 2020 and April 2023); and group 3 (post-pandemic, after May 2023); (Figure 1).

Exclusion criteria for this study were children at risk of severe RS virus infection and those with a history of premature birth, trisomy 21, or congenital heart disease.

### 2.2. Statistical Methods

To compare the characteristics of participants, the Kruskal–Wallis test was used for continuous variables and the chi-square test for categorical variables. Subsequently, clinical outcomes including the use of oxygen and/or mechanical ventilation were compared between the three groups, using logistic regression analysis. The time periods (groups) and age were used as covariates.

Statistical analyses were performed using Stata software version 15.1 (Stata Corp., College Station, TX, USA). Statistical significance was set at *p* < 0.05.

### 2.3. Ethical Approval

This study was approved by the ethics committee of Tokyo Metropolitan Toshima Hospital (approval number: Jin 6-5; date: 24 May 2024). The requirement for written informed consent was waived, owing to the use of anonymized data.

## 3. Results

### 3.1. Study Participants

**Figure 2:**
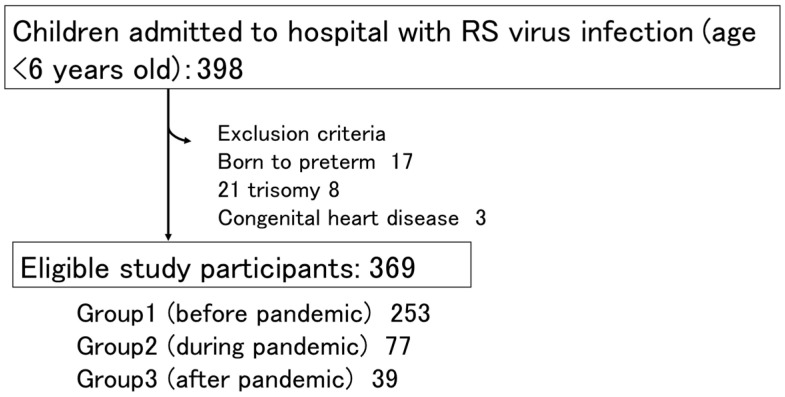
Flow chart of study participants.

During the study period, 398 children under six years old with RS virus infection were admitted to our hospital. Among these children, 17 were born preterm, 8 had trisomy 21, and 3 had congenital heart disease and were therefore excluded from the study. The final study cohort included 369 children comprising 253 in group 1 (pre-pandemic), 77 in group 2 (during pandemic), and 39 in group 3 (post-pandemic); (Figure 2).

### 3.2. Characteristics of Participants 

The clinicodemographic characteristics of the participants are presented in Table 1. The participants in group 2 were significantly older compared to the other two groups, whereas more boys were included in group 3. Laboratory data including white blood cell count, lactate dehydrogenase and C-reactive protein levels exhibited no significant differences between groups. Children with chest X-ray findings of pneumonia also showed no between-group differences.

### 3.3. Results of Logistic Regression Analysis

The results of the logistic regression analysis are presented in Table 2. The odds ratio (OR) for the use of oxygen was significantly higher in both groups 2 and 3 compared to that of group 1. Notably, the OR was 3.36 (95% confidential interval 1.59–7.12) in group 3. Additionally, age also exhibited a higher OR. The OR for using mechanical ventilation was higher in group 3 than in group 1. No significant differences were observed regarding age.

## 4. Discussion

The present study comparing the clinical features of children with RS virus infection before, during, and after the COVID-19 pandemic revealed the following results. First, children admitted to our hospital during the COVID-19 pandemic were significantly older and more required oxygen therapy compared to pre-pandemic admissions. More children admitted to our hospital after the COVID-19 pandemic required oxygen therapy and mechanical ventilation.

Previous research studies reported a reduction in the number of patients with RS virus infection due to the improved hygiene, sanitary measures, and travel restrictions [9], followed by increase in the number of children with RS virus infection [10]. In our study, we could not compare the numbers of infected children because this was a single-center study, but the older age in group 2 indicated that fewer younger children were exposed to going outside or were at nurseries. In fact, restrictions including nursery closure and travel restrictions were enforced during this period, and parents with younger children tended to avoid going out unnecessarily. This result is similar to that of a previous study that reported a higher age of children with RS virus infections during the COVID-19 pandemic [7]. Furthermore, our study revealed that the age of children with RS virus infection after the pandemic returned to the ages observed before the pandemic. The present study defined post-pandemic as the period during which COVID-19 was stated to no longer be a public health emergency of international concern, when restrictions were removed, and individuals began to move around like before the pandemic. Therefore, this result supports that these changes were a result of the non-pharmaceutical interventions that were implemented during the pandemic. 

Changes in disease severity can be attributable to viral factors or host factors. The virus genotypes were assessed, and no changes in RS virus-A or -B were observed [11], suggesting that changes in the severity of the clinical features were not a result of changes in the genotype of the RS virus. On the other hand, a reduction in RS virus antibody levels after the COVID-19 pandemic was reported [12]. Therefore, the increased severity of RS virus infection observed in children appears to be related to host factors. 

Among several factors associated with the severity of RS virus infections, the age of children is an important factor [13]. Although the most vulnerable population is under one year because, approximately, two-thirds of infants are reportedly infected with the RS virus during the first year of life [14]. However, as children aged under five years have medical visits associated with RS virus [15], we included children under six years of age and revealed that during the pandemic, children were older, yet more children required oxygen therapy. However, because of the pandemic, younger children were isolated during the period in which they would have normally been exposed to RS virus infection for the first time and might have therefore missed the usual timing for the first RS virus infection. In fact, no strict restrictions, such as during the COVID-19 pandemic, were implemented since the RS virus was first detected. As a result, changes in the epidemiology, such as a decrease in the number of children who experienced first-time infection before one year of age, is expected. This concept brings into question whether a delayed first infection may result in more severe symptoms, independent of the previously known age factor. 

Some limitations of the study need to be acknowledged. As this is a single-center study, patient selection may be inherently biased. However, we limited our investigation to children who were admitted, which may have minimized this bias to some extent. Further, clinical decisions were at the discretion of the attending physicians, without the implementation of standardized protocols. This could have also introduced bias into the results. To address such issues, a well-designed study with an appropriate clinical protocol is required.

Since the emergence of the COVID-19 pandemic, the concept of one health [16] has been highlighted, underscoring the potential for infectious diseases, including zoonoses, to pose a threat to another novel infectious disease and pandemic. Moreover, the increase in international travel has facilitated the easy spread of infectious diseases. Therefore, it is crucial to be aware of the implications and be adequately prepared for another impending pandemic. 

During the COVID-19 pandemic, while children exhibited fewer symptoms, the impact on their daily lives was greater than that of the adult population [17]. It is essential to understand the direct and indirect effects, such as the impact of other infectious diseases, in preparation for future pandemics.

## 5. Conclusions

The COVID-19 pandemic has altered the clinical features of RS virus infection. The age of children with RS virus infection was higher during the pandemic, and more children required treatment for more severe disease during and after the pandemic. 

## Figures and Tables

**Table 1:** Clinicodemographic characteristics of participants.

	Group 1 (n = 253) August 2017 to December 2019	Group 2 (n = 77) January 2020 to April 2023	Group 3 (n = 39) after May 2023
Age (years) (mean ± SD) ^1^	0.89 ± 0.79	1.24 ± 1.12 *	0.69 ± 0.65
Sex (male (%)) ^2^	141/112 (55.7)	39/38 (50.7)	30/9 (76.9) *
WBC (/mL) ^1^	10,247 ± 3717	10,302 ± 4859	11,546 ± 3873
LDH (IU/L) ^1^	333 ± 76	329 ± 75	318 ± 56
CRP (mg/dL) ^1^	1.62 ± 3.01	1.58 ± 2.73	3.79 ± 5.06
Chest X-ray (pneumonia: yes) (%) ^2^	74 (30.4%)	23 (33.8%)	16 (45.7%)
Use of oxygen (%) ^2^	113 (44.4%)	29 (49.1%)	26 (68.4%) *
Use of mechanical ventilation (%) ^2^	10 (4.0%)	2 (2.6%)	7 (25.0%) *

WBC, white blood cell; LDH, lactate dehydrogenase; CRP, C-reactive protein. SD, standard deviation. ^1^ Calculated using Kruskal–Wallis test. ^2^ Calculated using the chi-square test. * Significant difference

**Table 2:** Results of logistic regression analysis.

		Odds Ratio	95% CI	*p*-Value
Use of oxygen (yes/ no)	Group 1			
	Group 2	1.85	1.06–3.23	<0.05
	Group 3	3.36	1.59–7.12	<0.01
	Age	1.00	1.001–1.003	<0.001
Use of mechanical ventilation	Group 1			
	Group 2	0.75	0.16–3.52	0.71
	Group 3	4.89	1.71–13.94	<0.01
	Age	0.99	0.99–1.00	0.075

CI, confidence interval. Multiple logistic regression. Independent variables were the use of oxygen or use of mechanical ventilation.

## Data Availability

The datasets analyzed during the current study are available from the corresponding author upon reasonable request. Statistical analysis results are available from the publication by request to the corresponding author.
